# Mindfulness-based cognitive therapy for psychological distress in pregnancy: study protocol for a randomized controlled trial

**DOI:** 10.1186/s13063-016-1601-0

**Published:** 2016-10-13

**Authors:** Lianne M. Tomfohr-Madsen, Tavis S. Campbell, Gerald F. Giesbrecht, Nicole L. Letourneau, Linda E. Carlson, Joshua W. Madsen, Sona Dimidjian

**Affiliations:** 1 Department of Psychology, University of Calgary, Calgary, AB Canada; 2 Alberta Children’s Hospital Research Institute (ACHRI), Calgary, AB Canada; 3 Department of Pediatrics, University of Calgary, Calgary, AB Canada; 4 Department of Oncology, University of Calgary, Calgary, AB Canada; 5 Faculties of Nursing and Medicine (Pediatrics), University of Calgary, Calgary, AB Canada; 6 Alberta Children’s Hospital Research Foundation, Calgary, AB Canada; 7 Department of Psychology and Neuroscience, University of Colorado, Boulder, CO USA

## Abstract

**Background:**

Clinically significant psychological distress in pregnancy is common, with epidemiological research suggesting that between 15 and 25 % of pregnant women experience elevated symptoms of stress, anxiety, and depression. Untreated psychological distress in pregnancy is associated with poor obstetrical outcomes, changes in maternal physiology, elevated incidence of child physical and psychological disorders, and is predictive of maternal postpartum mood disorders. Despite the wide-ranging impact of antenatal psychological distress on mothers and their children, there is a gap in our knowledge about the most effective treatments that are available for psychological distress experienced in pregnancy. Additionally, no trials have focused on potential physiological changes that may occur as a result of receiving mindfulness training in pregnancy. The proposed trial will determine the effectiveness of an 8-week modified Mindfulness-based Cognitive Therapy (MBCT) intervention delivered during pregnancy.

**Methods:**

A randomized controlled trial (RCT) design with repeated measures will be used to evaluate the effectiveness of MBCT to treat psychological distress in pregnancy. A sample of 60 consenting pregnant women aged 18 years and above will be enrolled and randomized to the experimental (MBCT) or control (treatment as usual) condition. Primary (e.g., symptoms of stress, depression, and anxiety), secondary (cortisol, blood pressure (BP), heart rate variability (HRV), and sleep) and other outcome data (e.g., psychological diagnoses) will be collected via a combination of laboratory visits and at-home assessments from both groups at baseline (T_1_), immediately following the intervention (T_2_), and at 3 months postpartum (T_3_). Descriptive statistics will be used to describe sample characteristics. Data will be analyzed using an intention-to-treat approach. Hierarchical linear models will be used to test intervention effects on primary and secondary outcomes.

**Discussion:**

The trial is expected to improve knowledge about evidence-based treatments for psychological distress experienced in pregnancy and to evaluate the potential impact of mindfulness-based interventions on maternal physiology.

**Trial registration:**

ClinicalTrials.gov: NCT02214732, registered on 7 August 2014.

Protocol Version 2.0., 5 September 2016.

**Electronic supplementary material:**

The online version of this article (doi:10.1186/s13063-016-1601-0) contains supplementary material, which is available to authorized users.

## Background

Developmental trajectories in mental and physical health begin in utero; one of the earliest life experiences to confer risk to a developing fetus is prenatal exposure to maternal psychological distress [1]. Clinically significant distress in pregnancy is common, with epidemiological research suggesting that between 15 and 25 % of pregnant women experience an antenatal anxiety or mood disorder [2]. Similarly common are elevated levels of perceived stress and pregnancy-related anxiety (PRA) [3–7]. PRA refers to the extent to which pregnant women worry about their health, their baby’s health, labor and delivery, and caring for their new baby [7, 8].

A large body of research shows a relationship between maternal psychological distress and adverse maternal and child outcomes [9]. Maternal stress has been linked with preterm and low-birth-weight infants [4, 9–12], and unplanned caesarean delivery [13]. Antenatal psychological distress has also been shown to have a lasting influence on child health outcomes, showing associations with higher hypothalamic-pituitary axis (HPA) reactivity in response to stress, increased risk for sleep problems, and higher odds of developing inflammatory disorders, such as asthma and allergy, even after controlling for postnatal influences [14–18]. Exposure to antenatal psychological distress has also been linked to an increased risk of children developing anxiety disorders, attention deficit/hyperactivity disorder, conduct disorder, and cognitive deficits [19, 20]. The fact that psychological distress in pregnancy is unlikely to remit spontaneously [21] and is predictive of numerous negative impacts on the child and family [22–24] highlights the need for evidence-based interventions that effectively reduce psychological distress in pregnancy and prevent the emergence of clinically significant psychological distress in the postpartum period.

Despite the wide-ranging impact of antenatal psychological distress in infants and mothers, there is a gap in knowledge about the most effective treatments [25–28] and there have also been calls for further development and testing of interventions to manage psychological distress and anxiety in pregnancy [29, 30]. While there is a small research base using cognitive behavior therapy (CBT) and interpersonal therapy (IPT) during pregnancy to prevent postpartum depression, results have been mixed [30–32].

The majority of pregnant women with mental health concerns strongly prefer nonpharmaceutical treatments, citing concerns about the teratogenicity association with pharmacological intervention [26, 33, 34]. Matching individuals to their preferred treatment for mental health problems predicts higher levels of adherence, increased likelihood of entering treatment, and positive treatment results [35–38]. Previous studies have shown that pregnant women express a high degree of interest in Mindfulness-based Interventions (MBIs) for the treatment of psychological distress, and when they participate in the interventions they express high levels of engagement and satisfaction [9, 25].

### Mindfulness-based Interventions (MBIs)

MBIs are derived from Buddhist practice and focus on the cultivation of nonjudgmental awareness of present-moment experiences [39]. MBIs delivered in secularized interventions often stem from the Mindfulness-based Stress Reduction (MBSR) program, an 8-week, group intervention [40, 41]. MBIs have been adapted for the treatment of psychological distress associated with multiple disorders and illnesses in many populations [42]. Meta-analytic studies suggest that they produce moderate effect sizes for reductions of anxiety and/or mood spectrum disorders [43]. The time-limited, group-based, and nonpharmaceutical nature of MBIs makes them particularly promising options for interventions during pregnancy [25, 44]. Additionally, the scientific literature suggests that MBIs may be most useful for populations who are faced with chronic stress that requires active symptom management [43, 45]. The birth of a child, while joyful, also comes with significant and enduring adjustments for individuals and families, and sleep changes, and is accompanied by many unknowns that may contribute to feelings of worry or anxiety. The addition of a mindfulness-based acceptance strategy into this period of life could potentially serve to both reduce current experiences of distress and to prevent future distressing psychological symptoms.

Among the current evidence-based interventions, Mindfulness-based Cognitive Therapy (MBCT) was selected in this study because research suggests that it can effectively reduce current symptoms of stress and anxiety and may help in preventing recurrence of depression – an important consideration when working with a population of women whose antenatal psychological distress puts them at high risk for developing a postpartum mood disorder. MBCT was developed specifically to prevent relapse of depressive episodes. In populations of individuals with recurrent depression, participation in an MBCT program reduced the rate of relapse of depressive symptoms by approximately half of patients receiving standard treatment [46, 47]. Recent clinical trials have also examined the potential for MBCT to treat current symptoms of mild depression, anxiety, and stress and suggest that it successfully does so across multiple populations [46]. The MBCT intervention combines elements of MBSR with cognitive behavior therapy (CBT). The main goals of MBCT fall within three broad categories: cultivation of mindfulness (nonjudgmental awareness of present moment experiences), development of a positive, healthy attitudinal framework, and skill development for dealing with difficult moods [48].

### Mindfulness-based Interventions in pregnancy

Several studies have tested the efficacy and acceptability of MBIs delivered to pregnant women who are not currently reporting psychological distress or who report elevated levels of PRA – results suggest that MBIs increase positive affect and decrease symptoms of perceived stress, PRA, and depression [27, 33, 44, 49–51]. MBCT in pregnancy has been shown to reduce worry, anxiety, and comorbid symptoms of depression in pregnant women with clinically elevated symptoms of generalized anxiety disorder (GAD) [52]. In another study of pregnant women with a history of depression, participation in a modified version of MBCT (MBCT-PD; adapted for prevention of perinatal depression) led to a significant improvement in depressive symptoms [27]. One randomized controlled trial (RCT) of MBCT adapted for perinatal depression (MBCT-PD) showed significantly reduced rates of depressive symptoms and depressive relapse in women who received MBCT-PD compared to those who received treatment as usual (TAU) [53]. Although MBIs, specifically MBCT, have promise for the treatment and prevention of perinatal mood disorders, a recent systematic review on the impact of mindfulness and perinatal mental health concluded that there was “insufficient evidence from high-quality research” to make recommendations about the use of mindfulness to improve mental health during pregnancy [12]. Although recent work by Dimidjian et al. [53] demonstrated the effectiveness of MBCT-PD for reducing postpartum depression in a population of women with a history of depression, no studies have yet examined the efficacy of MBCT delivered to a population with a broader range of diagnoses, including high levels of pregnancy-related stress and anxiety, and none have examined the potential positive impact on physiological function.

## Potential physiological mediators of the effects of maternal psychological distress on birth outcomes and health

### Maternal psychological distress, cortisol, cardiovascular function, and sleep

Given the strong relationships between elevated maternal psychological distress in pregnancy and poor child outcomes, another question arises: How does maternal distress “get inside” the body of a growing fetus to influence developmental trajectories? One theory that may shed light on this question is the fetal programming hypothesis, which suggests that changes in the fetal environment (e.g., hormonal) can alter the structure and function of developing biological systems. There are several candidate mediators of the relationship between maternal psychological distress, birth outcomes, and later infant outcomes. One is the HPA, which is a primary pathway linking psychological and physiological experiences. Psychological states such as depression, anxiety, and stress have been associated with alterations in maternal HPA function (and subsequent changes in glucocorticoid levels), which is implicated in the transmission routes between maternal distress and infant development [14, 54, 55]. In the animal literature, fetal exposure to maternal stress is associated with an increase in basal levels of corticosteroids and an increase in corticosterone response to stress in the offspring [56]. Human studies suggests that in utero exposure to maternal psychological distress is also associated with alterations in the infant stress response – potentially impacting long-term HPA functioning and regulation of psychological and physiological responses to stressful events [57]. Maternal psychological distress has been associated with maternal cortisol levels during pregnancy [58–60] and exposure to elevated levels of maternal cortisol may link maternal distress and alterations in infant outcomes [61]. Hence, we are collecting information about cortisol, in order to assess the potential impact of MBCT on daily cortisol measures.

Cardiovascular stress reactivity is another potential mechanism linking maternal psychological distress in pregnancy and negative obstetrical outcomes. Cardiovascular stress reactivity naturally decreases as pregnancy progresses [62], which is thought to be an adaptive process that reduces the risk of experiencing gestational hypertension [63, 64]. However, reductions in cardiovascular stress responsivity are less likely to occur among pregnant women who report high levels of psychological distress. In fact, in women with high psychological distress in pregnancy, physiological reactivity to psychological distress has been shown to be of greater magnitude and duration, which increases the risk of significant vasoconstriction occurring during pregnancy [65]. Vasoconstriction in pregnancy can affect the uteroplacental blood flow and, furthermore, reduces the amount of oxygen and nutrients delivered to the developing fetus [66, 67].

Psychological distress has also been associated with disruptions in restorative physiological processes, such as subjective and objective measures of sleep [68–71]. Women experiencing high levels of psychological distress in pregnancy report significantly worse sleep quality, increased sleep disturbances, and higher daytime dysfunction [68]. Poor subjective sleep quality and objective measures of sleep, such as short sleep duration, have also been robustly associated with physiological markers of stress (e.g., cortisol and inflammation) [72, 73] and with negative obstetrical outcomes, such as elevated perception of pain and discomfort during labor, lengthy labor, and increased chance of requiring a caesarean delivery [74, 75]. Also, sleep disturbances during pregnancy are associated with negative birth outcomes such as preterm birth and low birth weight [73, 76]. Poor sleep in pregnancy may serve as another transmission route linking maternal psychological distress in pregnancy and poor obstetrical and infant outcomes. Therefore, we will examine potential changes in maternal subjective and objective sleep quality as a result of the intervention.

### Mindfulness and health

In addition to positively improving psychological symptoms, mindfulness has been associated with beneficial physiological changes. In healthy populations, trait levels of mindfulness have been associated with lower levels of circulating inflammatory proteins and reduced cortisol reactivity in response to a social stressor [77, 78]. MBIs have also been linked to reductions in psychological and physiological indices of stress arousal, [79] reductions in blood pressure (BP) and improved heart rate variability (HRV) [80, 81], and improvements in subjective sleep quality [41]. Theoretically, instruction in mindfulness could reduce maternal HPA reactivity in response to stress—and thereby reduce fetal exposure to glucocorticoids—reduce BP and BP stress reactivity, and improve sleep. These changes could translate into a more positive intrauterine environment for the developing fetus. The proposed research is innovative in that it combines fields of clinical and health psychology to evaluate not only the potential mental health benefits of MBCT but also the potential physical health benefits. The recent systematic review of MBIs in pregnancy suggested that researchers need to seek biological evidence of the effect of mindfulness, and that future studies need to test whether physiological pathways underlying the stress response change as a result of intervention [12]. The current study will build on previous research conducted in nonpregnant populations to investigate whether MBCT positively impacts important physiological variables, with known associations to birth outcomes and infant and child health.

### Three hypotheses

Primary hypotheses for the trial are:

1. When compared to a TAU control group, women who receive MBCT during pregnancy will, on average, experience lower symptoms of depression, worry, anxiety, and perceived stress over the course of the study

Secondary hypotheses for the trial are:

2. When compared to a TAU control group, women who receive MBCT will, on average, have lower levels of cortisol, lower BP, exhibit lower BP stress reactivity, have lower HRV and will have better sleep over the course of the study

Exploratory hypotheses for the trial are:

3. Initial levels of psychological distress will moderate findings, such that a larger treatment effect will be observed among women with higher psychological distress at baseline

## Methods

### Study design

The study is a two-arm, single-blinded, parallel-group RCT with equal allocation of participants and a repeated measure design. Table 1 illustrates the overall study design and process of enrollment, allocation, and follow-up of participants in the trial. The trial is being conducted at the Healthy HEARTS Laboratory, University of Calgary, Alberta, Canada. Ethics approval has been obtained from the University of Calgary, Conjoint Health Research Ethics Board (CHREB). Any trial amendments will be approved by CHREB before implementation and will be reported to the trail registry. A copy of the Standard Protocol Items: Recommendations for Interventional Trials (SPIRIT) checklist can be seen in Additional file 1.

**Table 1:**
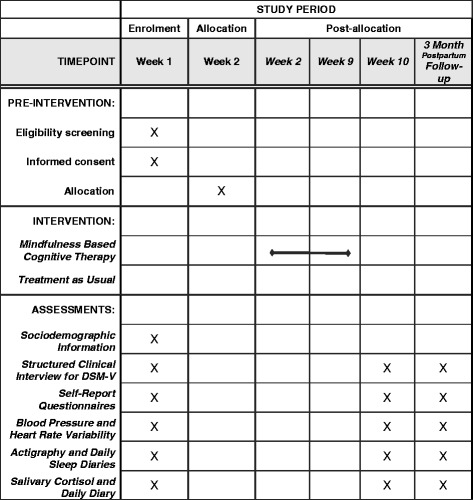
SPIRIT Schedule of enrolment, interventions and assessment

### Participants

Women who self-identify as experiencing high levels of psychological distress in pregnancy will be recruited through established recruitment sources including maternity clinics, posted announcements and pamphlets available in the main areas of family medicine clinics, midwifery clinics, holistic/chiropractic clinics, and other areas that pregnant women frequent (e.g., pregnancy-related trade shows, community centers, libraries). Participants will also be recruited through online advertising and media outreach.

### Inclusion and exclusion criteria

Eligible participants are women over the age of 18 years, who are between 12 and 28 weeks of gestation, with a singleton pregnancy, and who self-identify as experiencing high levels of psychological distress. High psychological distress is defined as a score of 4 or above on the Distress Thermometer – details about screening for psychological distress are presented below [82, 83]. Additional inclusion criteria include the ability to speak, read, and write English. There are several exclusion criteria: current use of antidepressant or anxiolytic medications; a history or current diagnosis of mental disorders with a psychotic, dissociative, hallucinatory, or delusional component; current major depressive disorder, current suicidality, current substance abuse or dependence; an inability to attend each of the classes or to participate in the assessments; and an unwillingness to be randomized.

### Screening, consent, and enrollment

During an initial telephone screening, a research assistant will describe the nature of the study, study protocol, and randomization process involved in the trial. If participants are interested in participating, inclusion and exclusion criteria will be assessed. During the initial telephone contact, the Distress Thermometer scale will be used to assess participants’ level of distress. The Distress Thermometer scale asks participants to rate their level of distress in the previous week on a 0 (not distressed) to 10 (extremely distressed) scale [84, 85]. Women who score 4 or above on the Distress Thermometer scale will be invited for further screening. Prospective participants who agree to be involved in the study will meet with a member of the research team who will explain the study procedures in full detail and obtain written consent. After consent has been obtained, participants will be administered a diagnostic interview (Structured Clinical Interview for the *Diagnostic and Statistical Manual of Mental disorders*, *fourth edition* (DSM-IV); SCID) to assess whether any exclusionary criteria are present [86].

### Baseline assessment

If no exclusionary criteria are met, participants will complete a battery of questionnaires assessing a host of demographic (e.g., age, gestational age, marital status, ethnicity, education, household income) and psychosocial (e.g., depressive and anxiety symptoms, positive and negative affect, worry, rumination, life satisfaction, and mindfulness) constructs. After questionnaires are completed, physiological measures will be obtained, including assessments of BP, heart rate variability (HRV), and a stress reactivity task. On the two weekdays immediately following the initial visit, participants’ salivary cortisol will be self-collected via oral swabs and an objective assessment of sleep quality will be obtained via actigraphy. All assessments occur within 2 weeks of participants being randomized to either an MBCT or a control group. All baseline measures will be collected before randomization occurs. A detailed description of all measures is presented below.

### Intervention

#### Mindfulness-based Cognitive Therapy group

The MBCT program takes place during weeks 2 to 9 of the study period. The intervention for the current study was adapted from the MBCT for perinatal depression (MBCT-PD) [27, 53]. Minor modifications were made to include a greater emphasis on managing stress and anxiety using mindfulness techniques – all information about preventing postpartum depression was retained [27]. The group intervention will be delivered in eight 2-h sessions. Goals of the program include helping women change their relationship to thoughts, feelings, and bodily sensations that can lead to psychological distress. Participants are guided to recognize and disengage from unhelpful mind states characterized by self-perpetuating patterns of ruminative thought. They also receive instruction in scheduling of mastery and pleasant events, and practice in mindful, assertive communication [27, 53]. A licensed clinical psychologist with training in delivering MBIs will lead the groups. Participants also receive a manual that includes relevant readings about the material being taught in sessions, a CD that includes guided meditations, and a DVD that demonstrates the mindful movement component of the class. The DVD includes demonstration of mindful movement poses by women at various stages of pregnancy.

#### Control condition

Participants in the TAU group are encouraged to engage in regular obstetrical and mental health care provided to them as part of their routine clinical care. All participants in the trial also receive an information pamphlet that contains contact numbers and website links for perinatal support services offered in Alberta. Participants are briefed with the information included in the pamphlet and informed about the perinatal support contacts should they choose to pursue treatment options.

#### Both groups

All participants who come to the laboratory will complete baseline (T_1_) measures prior to randomization. After enough participants have completed baseline assessments to form an adequately large MBCT group they will be randomly assigned using a block randomization procedure. Once all participants know their group assignment, they will be informed of their program start date by a study coordinator who is not involved in assessments. After randomization occurs, strategies will be employed to ensure that the assessors of outcomes remain blind to the experimental condition of all participants. All participants are invited back to the laboratory post treatment (T_2_) and at 3 months postpartum (T_3_) to complete the same measures. Data about any services received for management of mental health problems outside the study will be collected at each study assessment visit.

### Methods to protect against sources of bias

#### Randomization and allocation

The randomization process is not predictable, thereby reducing any potential for experimenter bias to influence participants’ allocation. The randomization scheme will be created by a random number-generating website (https://www.randomizer.org). The randomization sequence generation will take place prior to the recruitment of participants and will be completed by a Healthy HEARTS’ staff member not associated with the study, resulting in a completely random assignment of groups to the two conditions without investigator interference. Opaque envelopes will contain the randomization result for all groups. The envelopes are kept in a locked file cabinet to which assessors and research assistants do not have access.

#### Blinding

Because participants are not blind to group allocation, before each follow-up visit the study research coordinator will call to remind participants that the research assistants conducting the clinical interview and administering the physiological measures are unaware of group assignment, and should remain so. In order to further minimize the chance of unblinding, the research coordinator will remind the participants about blinding a second time when they arrive for their follow-up visits, and the research assistant conducting the diagnostic interview will also do so at the outset of that interview.

#### Simultaneous interventions

The intervention was designed to be an adjunct to TAU care for pregnant women experiencing psychological distress in pregnancy. As such, information about any engagement in adjunctive therapies will be collected at each follow-up point (T_2,3_) to control for this variable in subsequent analyses if it is found to impact primary or secondary outcomes.

### Schedule of visits

All participants complete self-report and physiological measures upon study entry (T_1_), 9 weeks later (T_2_), and at 3 months postpartum (T_3_). The duration of the TAU follow-up assessments is based on the time needed for the MBCT group to complete the intervention. Because the highest incidence of new-onset postpartum mood disorders occurs between 2 and 3 months after delivery, the third study time point is at 3 months postpartum [3, 87]. The control group will be offered the MBCT intervention following the trial (at 3 months postpartum). Questionnaire pilot data will also be collected as it pertains to the feasibility, acceptability, and effectiveness of offering MBCT in the postpartum period.

### Primary, secondary, and other outcome measures

#### Primary psychological outcomes

*The Pregnancy-Related Anxiety (PRA)* scale is a 10-item scale used to examine the extent to which women are worried about their own and their baby’s health, labor, delivery and caring for a new baby. Items are rated on a scale ranging from 1 (*never or not at all*) to 4 (*a lot of the time or very much*). The scale has shown strong associations with maternal and infant health outcomes over and above other traditional measures of state and trait anxiety [7, 88]. This scale has been found to have acceptable internal consistency (Cronbach’s alpha = 0.79).

*The Edinburgh Depression Scale (EDS)* will be used to assess symptoms of depression experienced during pregnancy and the postpartum period. Higher scores indicate more depressive symptoms. The EDS has been validated against interview schedules and other self-report instruments and has good internal reliability [89].

*The Generalized Anxiety Disorder Scale (GAD-7)* will be used to screen for and assess the severity of generalized anxiety disorder (GAD). Higher scores indicate more symptoms of GAD. The GAD-7 has excellent reliability and good criterion and convergent validity [90].

*The Perceived Stress Scale (PSS-10)* will be used to assess symptoms of perceived stress. The PSS-10 has good reliability and validity [91]. Among pregnant and postpartum women, it has been found to have a satisfactory level of internal consistency, ranging from 0.71 to 0.83 [92, 93].

#### Secondary physiological outcomes

*Salivary cortisol* will be collected at home on 2 weekdays using the following schedule: upon waking, 30 min after waking, and at 20:00 h. Participants will receive individualized training on collecting samples during their first study. They will be given instructions to use Salimetrics Oral Swabs (SOS) to collect saliva samples and an app (mEMA, developed by Tefsoft, Inc.) to answer questions about the timing of sample collection and their current mood. In addition, the importance of adhering to the schedule will be emphasized.

*Stress reactivity.* At each of the three visits (baseline, post-treatment and, follow-up), participants from both groups will complete two stress tasks: a mental arithmetic and a Stroop task [64]. Each task will be followed by a 5-min recovery period. After the first recovery period, researchers will take 2 min to explain the second task. During the baseline period, subjects will be fitted with cardiovascular recording equipment and will sit quietly for 5 min. During the stress tasks and recovery periods, participant’s HRV and BP will be continually assessed. At the beginning of each stress task condition, participants will be instructed to respond as quickly and accurately as possible without making errors. These tasks reliably induce a stress response (REF) and have been used reliably in pregnancy.

*Mental arithmetic task*. During this 3-min task, participants will be presented with a series of mathematical subtraction equations with the answers included on a computer screen. They will be asked to determine whether the answer to each equation is correct or incorrect. Each correct answer is followed by a beep emitted from a speaker. Each incorrect answer is followed by a noxious blare emanating from the same speaker. The task was designed to change in difficulty according to the participant’s ability to maintain a 60 % correct answer rate [94].

*Stroop task.* During the Stroop task, participants are asked to correctly identify the color of the stimulus on a computer screen [95, 96]. During the 5-min stress period, participants will see a series of words (“red”, “green”, “blue”, “yellow”) on the screen and will be asked to correctly identify the color of the stimulus (i.e., the text) by using one of the keyboard buttons. Each correct answer is followed by a beep emitted from a speaker. Each incorrect answer is followed by a noxious blare emanating from the same speaker. Each task is followed by a 5-min recovery period. Order of the stress tasks will be fully counterbalanced across participants.

*Blood pressure and heart rate variability* will be assessed after a 2-min resting period at baseline by taking the mean of three seated measurements obtained during a 5-min baseline period. Systolic BP and diastolic BP (in mmHg) will be obtained at 1-min intervals during a 25-min period that includes baseline, stress tasks, and recovery periods. BP data is collected using an automatic, calibrated, oscillometric BP monitor (BpTRU Vital Signs Monitor, BpTRU Medical Devices, Coquitlam, BC, Canada). Electrocardiography (ECG) (BIOPAC MP36 system and BIOPAC MP36 Student Laboratory Program, BIOPAC Systems, Goleta, CA, USA) will be used to collect heart rate data, which in turn will be used to calculate HRV. Heart rate will be monitored on a beat-to-beat basis during baseline, stress tasks, and the two recovery periods. Three ECG leads will be placed: two leads on either side of the upper chest, equidistant from the heart, and one lead on the left side of the mid-abdomen.

*Subjective and objective sleep*. Subjective sleep quality and quantity will be assessed using the Pittsburgh Sleep Quality Index (PSQI). The PSQI consists of 19 self-rated items and five questions rated by the roommate or bed partner. There are seven components of the PSQI including: subjective sleep quality, sleep latency, sleep duration, habitual sleep efficiency, sleep disturbances, use of sleeping medications and daytime dysfunction [97]. The PSQI has good reliability and its validity has been confirmed by concurrent polysomnographic findings [97] and among pregnant women [98]. Self-reported sleep hours often do not accurately reflect objective sleep time [99]; therefore, in this study participants’ sleep will also be assessed using wrist actigraphy (Actiwatch II, Philips, Pittsburgh, OH, USA). Participants will be asked to wear an actigraph on their nondominant arm for 48 h at each assessment point. Data will be collected on weekdays to minimize the variability caused by potential differences in weekday and weekend sleep patterns. In order to accurately assess rest and sleep intervals, participants will also complete a sleep diary and note the times when they go to bed, nap, and wake up during the day on which they wear the actigrapher. The Actiware Software (Version 6.0.1) will be used to configure and download the data from the Actiwatches. Assessment of sleep with actigraphy has demonstrated sensitivity to treatment effects [100].

#### Exploratory outcomes

We will also collect information about the diagnosis of axis I disorders of the DSM-IV (using the Structured Clinical Interview for DSM-IV; SCID) at each study visit [86]. The DSM-IV, text revision (DSM-IV-TR) includes standardized diagnostic categories and criteria for the classification of mental disorders [101].

#### Demographic assessment

Demographic information will include marital status, ethnicity, age, family socioeconomic status (household income, education level, neighborhood characteristics of the home), working status, and number of children in the home. Health practices will be assessed via self-report and will include smoking, alcohol consumption, and level of habitual exercise. Assessment of previous mental health problems and assessment of psychotropic medication use will be assessed via interview.

### Risks to the safety of participants

To our knowledge, there are no known risks to the pregnant women involved in the experimental (MBCT) or control (TAU) conditions of the study. There is a mindful movement component of the intervention, and in the first session a handout about safe exercise in pregnancy is reviewed with all participants. Stress testing has been conducted in pregnant women without adverse events [102]. If any adverse events occur (e.g., increased suicidal ideation reported to the study therapist or other project staff) we have a treatment algorithm in place to triage participants to appropriate acute or long-term treatments, as needed to ensure participant safety. Adverse events will be reported by the research coordinator to the University of Calgary Ethics Review Board using a serious adverse event report.

### Sample size

Based on the findings from a previous study, which examined the impact of a MBI during pregnancy on prenatal stress and mood, we expected a medium effect size of Cohen’s *d* = 0.5 [44] for the outcomes of PRA, anxiety, and depression for pre-post testing. Using a two-tailed test and a .05 significance level, 21 participants in each group (42 total) would provide adequate power (95 %) to reject the null hypothesis [103]. With an estimated attrition rate of 20 %, the number of participants required becomes a minimum of 26 participants per group (total *N* = 52).

### Strategies for assessing adherence

To assess adherence with the MBCT homework, we will ask participants to write down the amount of meditation practiced each day, in addition to the type of activity. Home practice records will be collected at the end of each session.

### Statistical analyses

Baseline characteristics (demographic and psychological) will be compared between groups using *t* tests (means) for continuous variables and chi-squared tests (%) for categorical variables to ensure randomization success. Any covariates found to differ significantly between groups will be held constant in between-group analyses. A significance level of .05 will be used for tests of all research hypotheses. The outcomes are between-group differences from baseline (T_1_) to post-intervention points (T_2,3_) on psychological and physiological measures. Analyses will be completed using Hierarchical Linear Modeling (HLM). HLM is a flexible approach to analysis of repeated measures data. For example, unlike analysis of variance (ANOVA), linear mixed models do not require that each participant has complete data to be included in the analysis, thus increasing their statistical power [104, 105]. All analyses will be completed using an intention-to-treat (ITT) approach—all participants will be included in the final statistical analysis according to the group (MBCT or TAU) to which they were randomized. To assess the effects of baseline psychological distress on outcomes, separate hierarchical mixed models of each outcome will be conducted using pretreatment psychological distress as a potential moderator.

### Day-to-day trial management

Dr. Tomfohr-Madsen (PI) will be responsible for overall project management. A full-time research coordinator has been hired and is responsible for the day-to-day trial management under the supervision of Dr. Tomfohr-Madsen. We have received ethics approval for the study and all materials, including questionnaires and consent forms, have been prepared. The MBCT protocol, as well as all handouts, audio-recordings, and videos were developed by Dr. Dimidjian’s team and have been modified to include information about stress and anxiety management. Currently, staff are hired and trained. Graduate students have been trained in the administration of the SCID and we have established an ongoing reliability team supervised by a licensed clinical psychologist. All data will be stored in a locked, secure area at the University of Calgary. Any paper work with patient identifiable data (e.g., consent forms, case notes, contact information) will be stored separately from anonymized data. Patient confidentiality will be protected through all phases of assessment, treatment, and data analysis in line with University of Calgary ethics guidelines. We expect to be have completed follow-up visits by April 2017.

### Access to data and dissemination policy

As the primary investigator on the trial, Dr. Tomfohr-Madsen, will remain the custodian of the data collected during the trial. Data will not be released by any third party (including the funder) before trial completion and will be analyzed independently by the study team. No interim analyses are planned and no interim data will be shared. Recognizing the importance of sharing results, data will be shared in accordance with the International Committee of Medical Journal Editors’ guidelines, which state that authors share with others the deidentified individual patient data underlying results presented in the trial reports (including tables, figures, and appendices or supplementary material) no later than 6 months after publication. Data will be made available upon request to the first author.

## Discussion

Perinatal mood disorders are highly prevalent, and their burden is significant. Clinically significant psychological distress in pregnancy is one of the best predictors of postpartum mood disorders, which are associated with numerous negative outcomes for the mother, the child, and the attachment relationship [24]. Depression and anxiety in pregnancy have also been associated with poor obstetrical outcomes and appear to predict infant health problems [22, 106] and mental health problems in children and adolescents, even after controlling for maternal postpartum mood. MBCT is a promising method for reducing symptoms of psychological distress and preventing the development of postpartum mood disorders [49–53]. Despite this, there are few rigorous examinations of MBCT in the perinatal period, and none to date that examine potential changes in maternal physiology.

Limitations of this study include the use of a TAU control group, whereby women enrolled on the trial are free to pursue services for the treatment of psychological distress experienced in pregnancy. It is also possible that women randomized to either group may seek out pharmacological and/or psychological interventions for the treatment of psychological distress and this may limit our ability to detect group differences between MBCT and the control condition. However, the decision to use a TAU control group was made due to ethical considerations, as we did not wish to discourage pregnant women from seeking treatment while experiencing active distress. In order to control for other treatment potentially sought by participants, we are collecting data about alternative types of treatment that women pursued while in the study. An additional limitation is that the group recruited will be a heterogeneous sample that will have varying levels of psychological distress and diagnostic profiles. While this methodological decision was made to enhance external validity, it may reduce internal validity. In spite of its limitations, this project is one of few aimed at assessing both the potential psychological and physiological changes associated with evidence-based mindfulness interventions delivered in pregnancy. The current study design also has a number of strengths, including: (1) interviewer-assessed clinical diagnosis of psychological disorders, (2) longitudinal study design with follow-up that extends into the postpartum period, and (3) extensive physiological testing, including assessment of BP, HRV, stress reactivity, HPA function, and sleep.

## Trial status

This study is recruiting participants.

## Additional file

Additional file 1: Includes a copy of the SPIRIT checklist. (DOC 122 kb)

## Abbreviations

ANOVA: Analysis of variance
BP: Blood pressure
CBT: Cognitive behavior therapy
DSM: Diagnostic and Statistical Manual of Mental Disorders
ECG: Electrocardiography
EDS: Edinburgh Depression Scale
GAD: Generalized Anxiety Disorder
HLM: Hierarchical Linear Modeling
HPA: Hypothalamic-pituitary axis
HRV: Heart rate variability
IPT: Interpersonal therapy
ITT: Intention-to-treat
MBCT: Mindfulness-based Cognitive Therapy
MBCT-PD: Mindfulness-based Cognitive Therapy adapted for perinatal depression
MBSR: Mindfulness-based Stress Reduction
MBI: Mindfulness-based Intervention
PRA: Pregnancy-related anxiety
RCT: Randomized controlled trial
TAU: Treatment as usual
